# Synthesis and production of steviol glycosides: recent research trends and perspectives

**DOI:** 10.1007/s00253-021-11306-x

**Published:** 2021-04-29

**Authors:** Marta Libik-Konieczny, Ewa Capecka, Monika Tuleja, Robert Konieczny

**Affiliations:** 1 The Franciszek Górski Institute of Plant Physiology, Polish Academy of Sciences, ul. Niezapominajek 21, 30-239 Krakow, Poland; 2 Department of Horticulture, Faculty of Biotechnology and Agriculture, University of Agriculture in Krakow, al. 29 Listopada 54, 31-425 Kraków, Poland; 3 Department of Plant Cytology and Embryology, Institute of Botany, Faculty of Biology, Jagiellonian University, ul. Gronostajowa 9, 30-387 Krakow, Poland

**Keywords:** Biotechnology, Diterpene glycoside, Secondary metabolism, Stevia

## Abstract

**Abstract:**

Steviol glycosides (SvGls) are plant secondary metabolites belonging to a class of chemical compounds known as diterpenes. SvGls have been discovered only in a few plant species, including in the leaves of *Stevia rebaudiana* Bertoni. Over the last few decades, SvGls have been extensively researched for their extraordinary sweetness. As a result, the nutritional and pharmacological benefits of these secondary metabolites have grown increasingly apparent. In the near future, SvGls may become a basic, low-calorie, and potent sweetener in the growing natural foods market, and a natural anti-diabetic remedy, a highly competitive alternative to commercially available synthetic drugs. Commercial cultivation of stevia plants and the technologies of SvGls extraction and purification from plant material have already been introduced in many countries. However, new conventional and biotechnological solutions are still being sought to increase the level of SvGls in plants. Since many aspects related to the biochemistry and metabolism of SvGls in vivo, as well as their relationship to the overall physiology of *S. rebaudiana* are not yet understood, there is also a great need for in-depth scientific research on this topic. Such research may have positive impact on optimization of the profile and SvGls concentration in plants and thus lead to obtaining desired yield. This research summarizes the latest approaches and developments in SvGls production.

**Key points:**

*• Steviol glycosides (SvGls) are found in nature in S. rebaudiana plants.*

*• They exhibit nutraceutical properties.*

*• This review provides an insight on different approaches to produce SvGls.*

*• The areas of research that still need to be explored have been identified.*

## Introduction

Steviol glycosides (SvGls) are chemical compounds of plant origin known for their unique property of sweetness that is much greater than that of sucrose. Since SvGls are also caloric-free, and have no harmful side effects on the human body, the use of these compounds as sweeteners is of great interest to scientists, food manufacturers and pharmacologists. An ability to synthesize SvGls has so far been described in a small number of plant species, the most famous of which being *Stevia rebaudiana* (Bertoni). Apart from *S. rebaudiana*, steviol glycosides have been found in three other species: *Stevia phlebophylla* (Kinghorn et al. 1984), *Rubus suavissimus* (Tanaka et al. 1981; Ohtani et al. 1992; Uhler and Yang 2018) and *Angelica keiskei* (Zhou et al. 2012). However, since SvGls content in these plants is rather low, they are of less economic importance than the steviol glycosides found in *S. rebaudiana*.

At least thirty-eight SvGls have been identified as typically present in stevia leaf extract (Ohta et al. 2010; Chaturvedula and Prakash 2011a, b; Chaturvedula et al. 2011; Ceunen and Geuns 2013a; Ceunen et al. 2012; Ibrahim et al. 2014; Montoro et al. 2013; Purkayastha et al. 2016). This, along with the fact that several health-promoting properties have also been attributed to other bioactive compounds of the herb (flavonoid glycosides, coumarins, phenolic acids, phenylpropanoids and some essential oil), has given *S. rebaudiana* Bertoni industrial and medicinal importance (Kim et al. 2011; Muanda et al. 2011; Lemus-Mondaca et al. 2012, 2016).

The best known and the most abundant SvGls in *S. rebaudiana* Bertoni are stevioside and rebaudioside A. Their concentration varies from 5 to 22% for stevioside and from 22 to 61.6% for rebaudioside A, depending on the genotype and cultivation conditions (Kennelly 2002; Ohta et al. 2010; Wölwer-Rieck 2012).

Since the approval of Stevia sweeteners in the USA by the FDA in 2008 and by the European Union in 2011, there has been an increase of industrial interest in these compounds (Stoyanova et al. 2011; González et al. 2014). Stevioside and rebaudioside A extracted from Stevia leaves are now more or less widely used in East and Southeast Asia and in South America, as a sweetener in a wide variety of foods.

Since the late 1970s, much research has been conducted on improving *Stevia rebaudiana* crop. The greatest concern for the breeders was the development of new varieties with characteristics appropriate for obtaining good quality raw material. However, the search is still on to find the right combination of genetically determined synthesis of steviol glycosides (in quantities needed by the food industry) or to identify novel, pharmaceutically relevant compounds and improvements in the efficiency of plant cultivation, including ease of obtaining propagating material, adaptation to environmental conditions and disease resistance.

The purpose of this publication is to summarize all the efforts made so far in relation to the SvGls acquisition. Knowledge in the field of biosynthesis of steviol glycosides and their function in plant physiology expand at high speed, while remaining fragmented and interdisciplinary. This makes it hard to keep up with state-of-the-art and be at the forefront of research. This is the main reason why a thorough and detailed review and evaluation of the source literature combined with advising the reader on the most pertinent and relevant research in biotechnology of SvGls is crucial. Furthermore, given the health-promoting properties of these chemicals and their possible impact on public health, there is also a need to encourage researchers to make efforts in this area by paving the way for further research.

## Steviol glycosides: biosynthetic pathway, chemical diversity and function

**Fig. 1:**
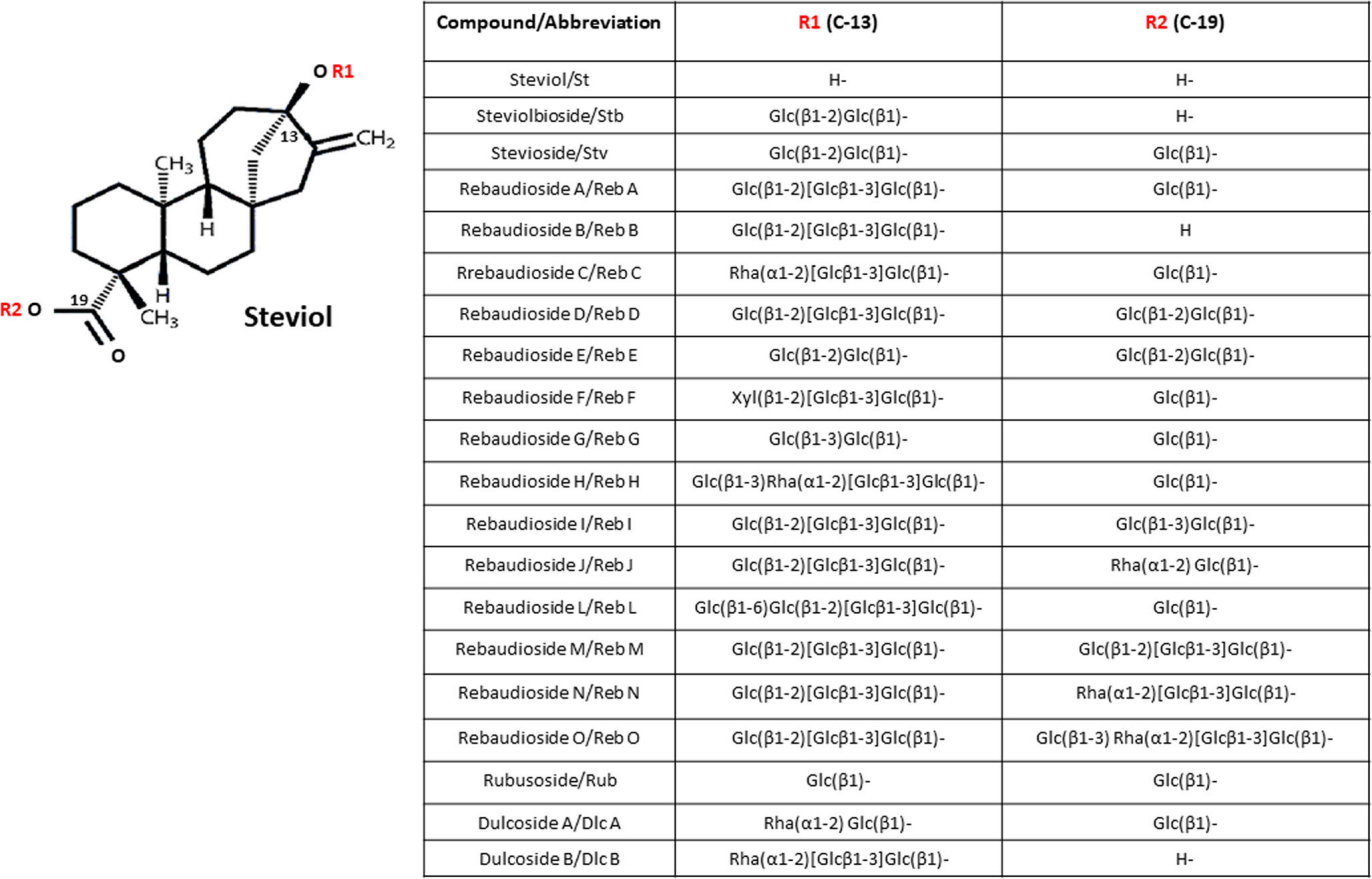
Chemical structure of some steviol glycosides. Glc, Rha and Xyl represent glucose, rhamnose, and xylose sugar moieties, respectively. Scheme prepared by authors, pictures of plastid and endoplasmic reticulum were created with BioRender.com

Steviol glycosides belong to the diterpenoid group of plant secondary metabolites. Their chemical structure is based on an aglycone core known as steviol (ent-13-hydroxyur-16-en-19-oic acid) to which a different number and types of sugar molecules are attached (Fig. 1).

**Fig. 2:**
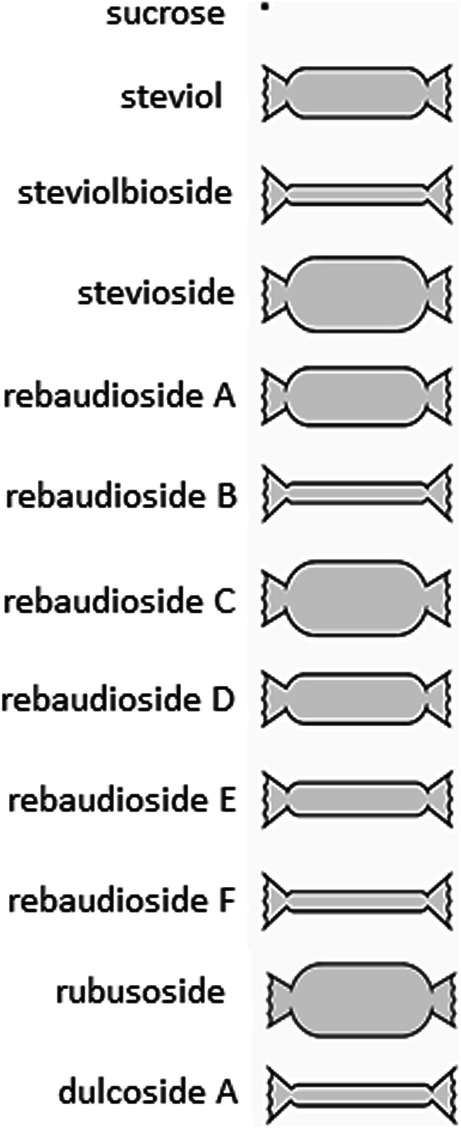
Comparison of the sweetness potential of the principal steviol glycosides found in stevia leaves (compared to sucrose). Scheme prepared by Agata Rębilas

The glycosylation of steviol at both C-13 and C-19 positions is responsible for the intensity of sweetness of SvGls. Both R1 and R2 glycone lengths and the number of total β-glycosyl residues correlate with sweetness (Spakman 2015). Moreover, Hellfritsch et al. (2012) demonstrated that replacing glucose with rhamnose reduces the sweet taste of steviol glycosides. Each of the steviol glycosides has its own unique taste and sweetness intensity (Fig. 2).

**Fig. 3:**
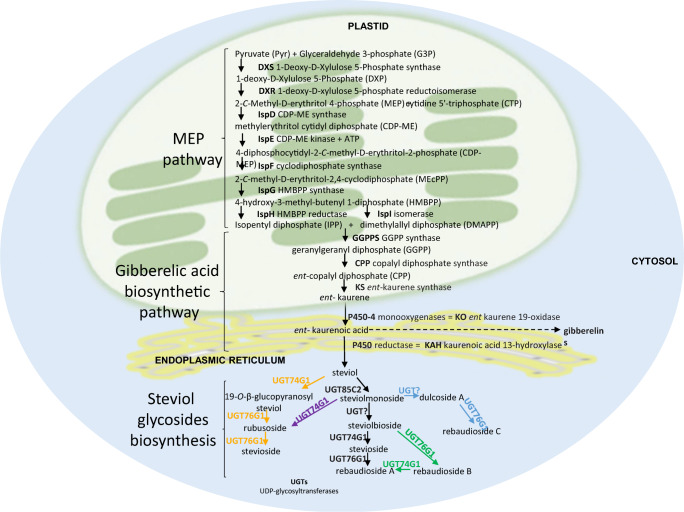
Schematic representation of steviol glycosides biosynthetic pathway with possible alternative routes (marked with different colours) of their biosynthesis. Scheme prepared by the authors

The SvGls biosynthetic pathway (Fig. 3) has previously been described by several authors (Brandle et al. 2002; Richman et al. 2005; Humphrey et al. 2006; Brandle and Telmer 2007; Mohamed et al. 2011; Kumar et al. 2012; Ceunen et al. 2012; Guleria and Yadav 2013a, b; Németh and Czinkόczky 2020); however, it still requires detailed research to better understand the regulation of this multi-step process. The initial steps of SvGls synthesis occur in the plastids following the multi-step methylerythritol 4-phosphate (MEP) pathway (Totté et al. 2000). During this phase, five-carbon building blocks, i.e. isopentyl diphosphate (IPP) and dimethylallyl diphosphate (DMAP) are formed from primary metabolic products, i.e. pyruvate (Pyr) and glyceraldehyde 3-phosphate (G3P). Next, the condensation of DMAPP with IPP leads to the production of geranylgeranyl diphosphate (GGPP), a building block for all terpenoids, including steviol and gibberellins (Zhao et al. 2013). Therefore, the SvGls and gibberellin biosynthetic pathways share several steps that lead to the conversion of GGPP by protonation initiated cyclization to ent-copalyl diphosphate (CDP) by CDP synthase (CPS). Next, CDP is converted into *ent*- kaurene by an ionization dependent cyclization catalysed by kaurene synthase (KS). *Ent*-kaurene then crosses the plastid membrane and is transferred to the endoplasmic reticulum, where it is oxidized to the ent-kaurenoic acid by a P450 monooxygenase, ent-kaurene oxidase (KO). Starting with the ent-kaurenoic acid, the biosynthesis of steviol deviates from that of gibberellin. Situated at an important branching point, the ent-kaurenoic acid can be hydroxylated at C-7 to ent-7α-hydroxykaurenoic acid and further to the gibberellins, whereas hydroxylation at C-13 catalysed by the kaurenoic acid 13-hydroxylase (KAH) leads to steviol. After the formation of steviol, a series of glycosylations takes place in the cytosol, leading to the production of different SvGls. These reactions are catalysed by the cytosolic UDP-dependent glycosyltransferases (UGTs). They transfer a sugar residue from an activated donor (mostly UDP-glucose) to an acceptor molecule. It has been suggested that glycosylation of steviol by UGT85C2 leading to the production of steviolmonoside is a key regulatory step of steviol glycoside biosynthesis (Mohamed et al. 2011). Further steps of the glycosylation pathway are known to occur mainly in planta. They involve glycosylation of steviolmonoside to form steviolbioside by unknown UGT, followed by UGT74G1-catalysed glucosylation of steviolbioside that yields stevioside, and next, UGT76G1-catalysed glucosylation of stevioside to form rebaudioside A. Further glycosylations also take place as evidenced by the presence of SvGls containing up to seven glycosyl groups (rebaudioside O). So far, the complicated pathway of glycosylation has not been fully understood. Rhamnosyl- and xylosyltransferases have not been described, leaving incomplete biosynthesis of, e.g. dulcoside A and rebaudioside C or rebaudioside F (Ceunen and Geuns 2013a). In addition, studies performed on transgenic plants have revealed the wider range of potential biosynthetic routes that lead to the formation of rubusoside, rebaudioside B and rebaudioside C (Guleria and Yadav 2013a; Kim et al. 2019).

The final phase of SvGls accumulation is the translocation of glycosylated steviol into the vacuole. However, the transport mechanism into this organelle has not been explained yet.

The extensive transcriptome research has led to the identification of several potential genes involved in the biosynthesis pathway of SvGls. The use of an expressed sequence tags (ESTs) technology has led to identification and characterization of three candidate UGTs involved in the glucosylation from steviol to rebaudioside A (SrUGT85C2, SrUGT74G1, SrUGT76G1) (Richman et al. 2005; Brandle et al. 2002). Next-generation sequencing (NGS)-based RNA-Seq technology was used to identify a total of 143 unigenes as glycosyltransferase, but none of them have been functionally characterized (Chen et al. 2014). A reconstruction of biosynthetic pathway for the production of SvGls in *Escherichia coli* and in *Saccharomyces cerevisiae* and subsequent RNA-Seq analysis allowed for an identification of a novel 13α-hydroxylase (KAH_ACD93722) and a UDP-glucosyltransferase UGT91D2w with the activity of steviol-13-monoglucoside-1,2-glucosyltransferase as well as novel cytochrome P450 enzymes: kaurene oxidase (KO75) and kaurenoic acid hydroxylase (KAH82) (Wang et al. 2015, Gold et al. 2018, Moon et al. 2020). The studies concerning genes expression engaged in SvGls biosynthesis during different developmental stages of *S. rebaudiana*, using cDNA libraries sequenced with Illumina GAIIx platform, have indicated developmental phase-dependent regulation of key gene expression and transcription factors (Singh et al. 2017). Moreover, 124 cytochrome P450 monooxygenases and 45 UGTs have been identified as potential targets in plant engineering to upscale SvGls biosynthesis and to implement molecular breeding strategies to enhance the plant genetically. Recently, full-length *S. rebaudiana* transcriptome data has been generated using a single-molecule, real-time, long-read sequencing (SMRT) technology known as the third-generation sequencing platform (Zhang et al. 2020). This study could serve as a valuable resource for future research into *S. rebaudiana* and could also be of benefit to studies in other closely related species.

The function of SvGls is well established, and the benefits for the human health are unquestionable. There have been many studies on the possible effects of stevia extracts or individual steviol glycosides as antidiabetic drugs (reviewed by Gupta et al. 2013; Abdel-Aal et al. 2021). The obtained data have demonstrated that the compounds derived from stevia provide a comprehensive set of mechanisms counteracting the mechanics of type II diabetes and its possible complications.

The question remains of what benefits the presence of large amounts of these secondary metabolites in *S. rebaudiana* brings for the plant itself, especially given the significant metabolic cost. Ceunen and Geuns (2013a) presented several hypotheses about SvGls function in a plant. They have pointed to steviol as the most bioactive compound that could act as a gibberellin precursor; however they have also mentioned several studies that contradict this hypothesis. A possible function of SvGls synthesis in a defence mechanism against insects has been also discussed by Ceunen and Geuns (2013a); however it remains the hypothesis inconclusive. The authors have also suggested that SvGls might serve as a long-term energy reserve, e.g. to satisfy the energy demands during flowering and seed ripening. Finally, they have indicated that SvGls might play a role in the cellular antioxidant network since they have the capacity to act as potent scavengers of reactive oxygen species (ROS). In vitro assays with purified steviol glycosides revealed excellent scavenging activities against hydroxyl radicals (Stoyanova et al. 2011). The differences seen between antioxidant potential of stevioside, rebaudioside A, and rubusoside might indicate that their antioxidant activity is mostly related to their common diterpene skeleton.

## ***Stevia rebaudiana*** (Bertoni) biology and agronomy

*Stevia rebaudiana* belongs to the *Asteraceae* family and originates from subtropical region in North Eastern Paraguay (22–24° S). It can be found on grasslands and at the edges of marshes with infertile sand or marshy soils with shallow ground water level (Madan et al. 2010). The climate there is semi-humid subtropical, with temperatures ranging from − 6 to 43 °C with an annual average of 23 °C and rainfall of 1500–1800 mm a year.

**Fig. 4:**
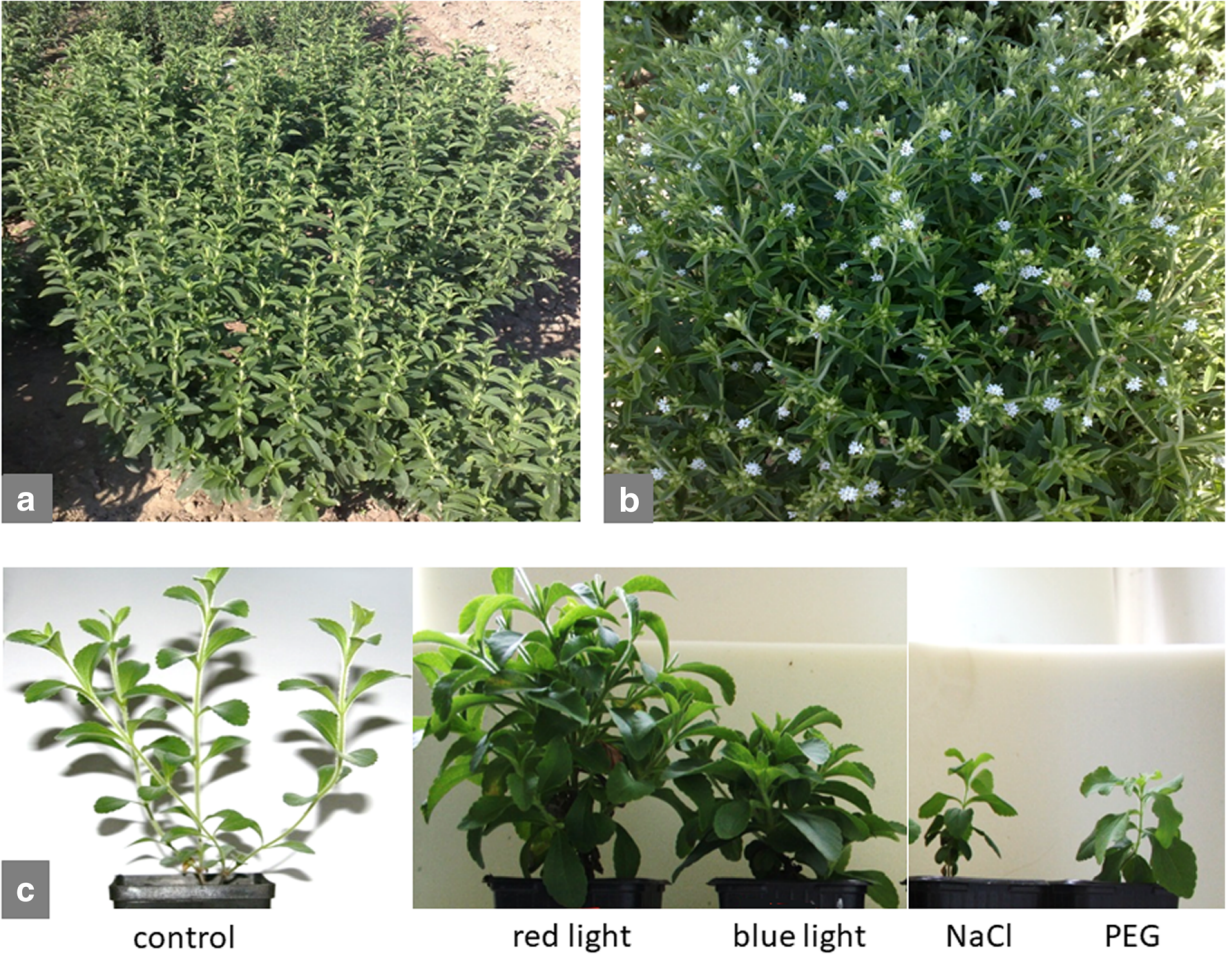
Morphology of *Stevia rebaudiana* plants at full vegetative growth (**a**) and at the beginning of flowering (**b**) cultivated in the open field under temperate climate conditions of Southern Poland or cultivated in the growth chamber under influence of different stress inducing factors (**c**) (source: unpublished photos by Marta Libik-Konieczny)

*S. rebaudiana* is a herbaceous plant, grown as a perennial in the subtropical regions, including Mediterranean, and as an annual plant at middle to high latitudes (Brandle et al. 1998). The plant forms an extensive, relatively shallow root system and several upright stems that branch as growth progresses. In cultivation plants may reach above 1 m in height, but usually they reach up to 60–70 cm (Fig. 4a). Leaves are sessile, opposite, elliptical to lanceolate, more or less serrated at the edges of the tips. Whole shoots are slightly pubescent (Madan et al. 2010). Tiny white flowers, typical of *Asteraceae* morphology, are gathered in numbers from 2 to 6 in small capitula arranged in loose panicles (Fig. 4b). The fruit — achenes with pappus bristles — is slender about 3 mm in length. As a short-day plant, *S. rebaudiana* flowers from January to March in the Southern hemisphere and from September to December in the Northern hemisphere. Long-day conditions prolong vegetative growth, significantly increasing leaf biomass. Stevia is a self-incompatible, but highly cross-pollinated plant. This is the reason for the high variability of generatively multiplied progeny. In addition, seeds are characterized by poor viability. In mid-longitude regions, due to the photoperiodic sensitivity of stevia, seed production is difficult because of the flowering at short days, when low temperatures prevail and limit fruit ripening (Gantait et al. 2018).

**Fig. 5:**
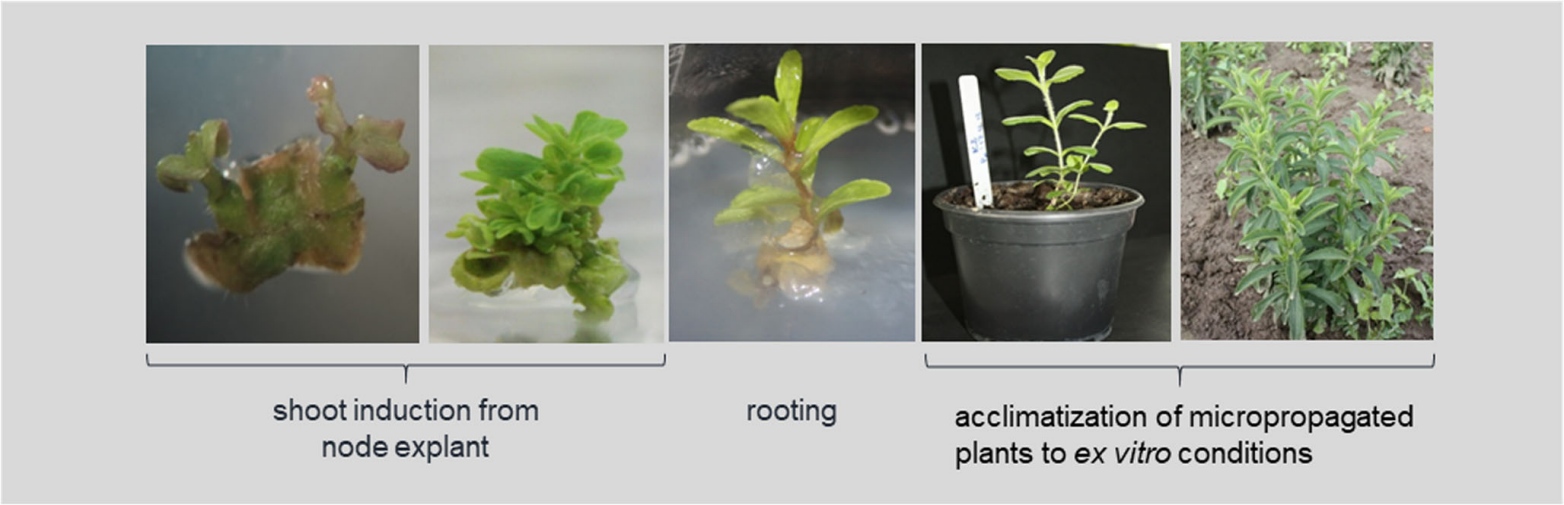
Following steps of *Stevia rebaudiana* micropropagation. (source: unpublished photos by Marta Libik-Konieczny)

The most common composition of SvGls in the wild variety of *S. rebaudiana* includes stevioside (5–10%); rebaudiosides A (2–5%) and C (1%); dulcoside A (0.5%); rebaudiosides D, E, and F (0.2%); and steviolbioside (0.1%) (Ceunen et al. 2012). Bondarev et al. (2003) indicated that the greatest content of total SvGls is noted in leaves, followed by that in flowers, next stems, then seeds and finally roots. Following the study by Sekaran et al. (2007), Pande and Gupta et al. (2013) reported a slightly different order of declining SvGls content, i.e. leaves > shoots > roots > flowers (Fig. 5a). According to Lavini et al. (2008), the total content of SvGls in flowers and stems is, respectively, 7–8 and 12–13-fold lower than in leaves, while in seeds 2–2.5-fold lower than in flowers. During ontogenesis, a gradual increase in SvGls content in mature shoots is observed, which continues until flower buds are formed and flowering begins (Bondarev et al. 2003). However, a significant decrease is observed in the SvGls content in mature stevia leaves during flowering (Ceunen and Geuns 2013a).

Besides variations in total SvGls level, their individual composition can also vary significantly, depending on genotype, ontogeny, and photoperiod. Various plant types with larger amounts of specific glycosides and several cultivars of better plant yield have already been released and/or patented around the world, including such countries as Korea, Japan, Indonesia, China, the USA, Canada, Taiwan, Russia and India (Ramesh et al. 2006; Yadav et al. 2011). Lemus-Mondaca et al. (2012) reported on about 90 varieties of *S. rebaudiana*.

*S. rebaudiana* is reported to be more vigorous when cultivated as a crop than in its native habitat, and since it has an immense adaptability to various environmental conditions, it has been successfully introduced to many countries around the world. Information on the cultivation of *S. rebaudiana* is available on regions from the equator (Indonesia), through Japan, Brazil, Korea, Mexico, the USA, Tanzania and India, to the northern latitudes 50–60° (Canada, Poland, Russia) and the southern 30° (Argentina) ones. At present, production of stevia has emerged mainly in China, while the primary market is in Japan (Brandle et al. 1998; Ramesh et al. 2006; Kim et al. 2011; Aladakatti et al. 2012; Kąkol et al. 2014; Gantait et al. 2015; Libik-Konieczny et al. 2018; Ciriminna et al. 2019). *Stevia rebaudiana* does not have high soil requirements, but a uniform moisture supply and good drainage should be provided. Full access to light is crucial, because even partial shade results in slower growth, delayed flowering, lower yield of leaves and content of SvGls (Ramesh et al. 2006).

Properly planning enables up to 8 years of cultivation with up to six harvests a year (Lemus-Mondaca et al. 2012). In India, intensive growth with increased plant density and regular irrigation produced 8–11 t ha^−1^ of dry leaves from five cuts per year (Aladakatti et al. 2012). In northern Italy, the cultivation with two cuts per year lasted 8 years, while in the southern region they gave good yields (3.3–4.6 t ha^−1^) for 2 years (Lavini et al. 2008). Further north cultivation of *S. rebaudiana* is possible only during one growing season. Brandle and Rosa (1992) obtained about 2.1–3.5 t ha^−1^ in Canada. In Poland, field growing was successful between mid-May and the end of September when a single harvest gave the yield of dry leaves ranging from 1.6 to 3.3 t ha^−1^ (Kąkol et al. 2014). These results indicate that leaf yields in mid-latitude regions did not differ from the average yields recorded in the areas of subtropical climate. The need to produce plant material every year requires, however, an establishment of a reliable farming process. For this reason, these regions cannot compete in large-scale production of SvGls.

*S. rebaudiana* plants are conventionally propagated through cuttings, but this traditional method cannot produce a large number of plants. It also demands a large number of mother plants and carries the risk of transferring diseases to the crop. Since the seeds of *S. rebaudiana* are characterized by a very low percentage of germination, thus raising seedlings by sexual plant reproduction is also limited (Aman et al. 2013). Therefore, plant material obtained through in vitro culture is used most often to grow *S. rebaudiana.* It creates a potential for a rapid mass production of planting material (Yadav et al. 2011; Pande and Gupta 2013; Yücesan et al. 2016; Rosales et al. 2018). Although it is very labour and cost-intensive, it results in a cultivation of plants with desirable characteristics. And while it requires more investment, in the long run it brings better economic effects, especially when the crop is to be free of pathogens and yield crops for several years (Das et al. 2010). In vitro propagation is not only an important alternative to the conventional propagation of the crop but also to the procedures used in breeding.

## Conventional approaches to steviol glycosides production

A growing demand for plant material for SvGls production is associated with and increased cultivation of *S. rebaudiana* all over the world. However, this recently developed crop suffers from a lack of high-value adapted and traceable cultivars.

### Cultivars selection

Recently, Angelini et al. (2016) have listed ninety cultivars of *S. rebaudiana*. These genotypes, in which important traits such as total glycosides, rebaudioside A, and rebaudioside A/stevioside ratios were enhanced, have been mainly bred through mass selection. Agricultural practices show that among all these cultivars, “Eirete”, “Criolla” and “Morita” are the most frequently cultivated. A new cultivar, *S. rebaudiana* Morita, is known to produce an extremely high ratio (60% ) of total rebaudioside A, which is known to induce an optimal taste of sweetness (Ohta et al. 2010).

### Polyploidy induction

One of the promising approaches to improve steviol glycosides yield in *S. rebaudiana* might be polyploidy breeding. Induction of polyploidy by antimitotic agents application is mainly used to increase the plant’s biomass; however as a consequence, it can lead to enhancement of secondary metabolites production (Gantait et al. 2020). There have not been yet a sufficient number of experiments in *Stevia rebaudiana*; however, it is known that occasionally appearing mixoploidy plants express an increase in stevioside content (Hegde et al. 2015). Moreover, it has also been shown that the tetraploid *S. rebaudiana* plants exhibit higher content of stevioside and rebaudioside A than the diploid controls (Zhang et al. 2018). These tetraploid plants may be further selected for breeding purposes or micropropagated for commercial production.

### Abiotic and biotic factor manipulation

Secondary metabolites are compounds that are not directly involved in plant growth and development, but they are involved in the interaction of a plant with its environment (Gantait et al. 2021). Therefore, it can be assumed that SvGls representing a type of secondary metabolites in *S. rebaudiana* may provide protection against biotic and abiotic factors. A number of studies strongly suggest that a wide range of environmental factors such as day length, quantity and quality of light, temperature, drought and salinity stress, nutrients availability and arbuscular mycorrhysis affect SvGls contents in *S. rebaudiana* plants significantly (Table 1).

**Table 1:** The examples of conventional approaches for steviol glycoside synthesis

Technique		SvGls content	References
Cultivation in growth chamber	Photoperiod/ontogenesis	Accumulation of SvGls in upper leaves significantly higher than in lower leaves under both LD and SD conditions. Reb A to Stv ratio increased significantly in upper leaves under SD conditions	Mohamed et al. 2011
Cultivation in greenhouse	Photoperiod/ontogenesis	Long day significantly increase SvGls content. Higher ratio of Reb A to Stv is found during vegetative stages of plants under short day	Ceunen and Geuns 2013b
		No significant changes under the short-term treatments of different photoperiods in SvGls contents, and the transcript levels of genes involved in SvGls biosynthesis in fast growing period while their significant upregulation both in flower-bud appearing stage and flowering stage	Yang et al. 2015
Cultivation in growth chamber	Photoperiod/long night interruption by red LED light	SvGls increase	Ceunen et al. 2012
	Red/far-red light-emitting diodes (LEDs) and blue LEDs	Increase in total SvGls content and transcriptions activity of some genes involved in SvGls biosynthesis. SvGls content was higher in blue light than that of other light treatments	Yoneda et al. 2017
Cultivation in greenhouse	Temperature and dehydration	Maximal transcriptional activity of several genes involved in SvGls biosynthesis at 25 °C, and inhibition of their activity at both low temperature (15 °C) and high temperature (35 °C). Downregulation of investigated genes in leaves of dehydrated plants	Yang et al. 2015
Cultivation in greenhouse	Drought stress induced by PEG	Downregulation of several genes involved in SvGls biosynthesis, significant decrease in the amount of total and particular SvGls	Hajihashemi and Geuns 2015
cultivation in growth chambers	Salinity stress	Decrease in Reb A and Stv content in high salinity stress	Zeng et al. 2013
	Salinity stress	No effect of low salinity stress on Stb and Reb A content, decrease in Stv and Reb A under higher level of salinity stress	Cantabella et al. 2017
Cultivation in the field	Salinity stress	Increase of SvGls content in low salinity stress	Shahverdi et al. 2019
Cultivation in the field	Exogenous supply of plant nutrition	SvGls increase	Pal et al. 2013; Benhmimou et al. 2018
Cultivation in the field	Plants inoculation with arbuscular mycorrhizal fungi	Upregulation of transcriptional activity of several genes involved in SvGls biosynthesis	Mandal et al. 2015
Cultivation in the field	Controlled elicitation with salicylic acid, chitosan and hydrogen peroxide	SvGls increase due to elicitors (especially salicylic acid), inducement of gene expression-associated to the biosynthesis of SvGls	Vazquez-Hernandez et al. 2019

#### Changes in photoperiod

Studies of the effect of photoperiodism on the accumulation of SvGls showed that under long-term conditions, vegetative growth is prolonged, significantly increasing the leaf biomass and the total content of SvGls (Ceunen and Geuns 2013a, b). However, Yang et al. (2015) unexpectedly found no significant changes in the activity of several genes involved in SvGls biosynthesis in plants exposed to different photoperiods. On the other hand, a clear influence of the stages of stevia growth on the gene transcripts level and SvGls content has been observed along with their significant increase during the flower bud formation phase and flowering phase. It has also been noted that SvGls accumulates more in the upper leaves than in the lower leaves under both long day and short day conditions, while the short day leads to a significant increase in the rebaudioside A to stevioside ratio in the upper leaves (Mohamed et al. 2011). These findings may be of particular interest when harvesting suitable plant material for the SvGls extraction. Ceunen et al. (2012) have developed a simple procedure to sustain the vegetative growth as well as the accumulation of SvGls in the leaves of plants growing in short photoperiods by long night interruption by red LED light. They have found a 55% increase in SvGls content in groups of plants growing in red LED light interrupted by long night conditions compared to control plants cultivated under short day conditions.

#### Changes in the light quality

Another approach that could serve as a practical way to increase SvGls content is using specific light treatment during plant cultivation. Yoneda et al. (2017) have efficiently modulated SvGls content and transcriptional activity of several SvGls biosynthesis-related genes by changing supplemental light and by growing plants under red/far-red light-emitting diodes and blue light-emitting diodes. They have detected a higher transcription level of *UGT85C2*, a gene involved in catalysing the sugar-transfer reaction under both treatments together with significant increase in rebaudioside A and stevioside content. Moreover, the influence of light quality on plants morphology has been noted and shown to be particularly significant in stevia plants under blue light treatment causing plants to have shorter stems than control or red/far-red light treated plants. In our studies, we have also observed a similar tendency (Fig. 4c).

#### Temperature manipulation

The influence of temperature and water availability on SvGls content and the activity of genes involved in biosynthetic pathway of SvGls have been studied by Yang et al. (2015). Their observations show that the transcript level of fifteen genes reach maximum at 25 °C, while the transcriptions of some genes are restrained both in low temperature (15 °C) and high temperature (35 °C).

#### Application of drought and salinity stress during cultivation

Drought stress, simulated by the application of polyethylene glycol in different concentrations 5–15%, had a negative effect on plant growth and the content of SvGls and transcriptional activity of some genes from SvGls biosynthetic pathway (Hajihashemi and Geuns 2016). In our studies we have also observed a significant decrease in stevia plant height when treated with 1 mM PEG solution (Fig. 4c). Therefore it can be suggested that sufficient irrigation during stevia cultivation is needed to obtain a high yield of plant material with high content of SvGls.

Studies concerning the effect of salinity stress on *S. rebaudiana* growth and production of SvGls have led to contradictory results. Shahverdi et al. (2019) have suggested that the application of a low salinity level (30 mM NaCl) has a stimulatory effect on chlorophyll a, carotenoids, total sugar content as well as the percentage of stevioside and rebaudioside A, although it decreases plant growth. Similar results have been found in our preliminary studies with an application of 45 mM NaCl during stevia plant growth. A significant decrease in plant height has been observed (Fig. 4c). Zeng et al. (2013) have found that 90–120 mM NaCl treatment of stevia plants notably decreases the content of rebaudioside A and stevioside; however lower salinity stress does not influence plant growth parameters. The decrease in plant height and dry weight has been observed only under higher levels of salinity (120 mM), indicating that *S. rebaudiana* is moderately tolerant to salt stress. On the other hand, the studies performed by Cantabella et al. (2017) with application of 85 mM NaCl (5g/l) have indicated that salt stress has no significant effect on plant growth and stevia plants exhibit physiological adaptation that allow them to cope with NaCl-induced oxidative stress, and a particular SvGls profile may be involved in this adaptations.

#### Nutrient supplementation

Studies concerning optimum nitrogen, phosphorus and potassium levels needed for higher dry leaf yield and steviol glycosides content in *S. rebaudiana* plants (Benhmimou et al. 2018) have led to a conclusion that although stevia is thought of as low to moderate nutrition requiring plant (since this crop can adapt to poor quality soils), the necessity of fertilizers application may vary and depends on the environment and soil type.

#### Mycorrhization and elicitors application

Arbuscular mycorrhizal symbiosis of *S. rebaudiana* plants inoculated with *Rhizophagus intraradices* led to upregulation of eleven genes involved in SvGls biosynthetic pathway (Mandal et al. 2015). It is a result of improved nutrition and enhanced sugar concentration due to increased photosynthesis in mycorrhizal plants. Similarly, Vazquez-Hernandez et al. (2019) have demonstrated that controlled elicitation of stevia cultivation might be used to improve the yield of SvGls. Application of different elicitors such as salicylic acid, chitosan and hydrogen peroxide leads to an increase in the number of leaves in the stevia plant and to an enhancement in SvGls content, correlated with an induction of transcriptional activity of several genes engaged in biosynthesis of these compounds.

These approaches carried out in field or greenhouse conditions are associated with the risk of less uniformity of achieved plant material due to a significant genetic variability in SvGls production (Modi and Kumar 2018; Bogado-Villalba et al. 2020). Considerable levels of variance are seen not only between plants of the same cultivar, but even between similar plants in the same developmental stage. For example, in a field study with 300 *S. rebaudiana* plants, total steviol glycoside content varied between 0.5 and 3.7% w/w at the seedling stage and between 6.7 and 18.6% w/w at harvest time (Nakamura and Tamura 1995).

## Biotechnological approaches to steviol glycosides production

Several biotechnological attempts have been made so far to enhance SvGls production in plant material in in vitro conditions or to produce these compounds de novo (Tab. 2).

**Table 2:** The examples of biotechnological approaches for steviol glycoside synthesis

Technique		SvGls content	References
Micropropagation		No effect	Kumari and Chandra 2015
		SvGls decrease	Bondarev et al. 2001
Micropropagation + elicitors	alginate and yeast extract	SvGls increase	Bayraktar et al. 2016
Micropropagation + salinity stress	NaCl	Upregulation of several genes encoding key enzymes of the steviol glycoside biosynthetic pathways and SvGls increase	Pandey and Chikara 2015
	NaCl, Na_2_CO_3_	SvGls increase	Gupta et al. 2016
	NaCl	SvGls decrease	Fallah et al. 2017
	NaCl	SvGls decrease	Rameeh et al. 2017
	Glycine betaine	SvGls increase	Rameeh et al. 2017
	NaCl	Upregulation of several genes encoding key enzymes of the steviol glycoside biosynthetic pathways	Lucho et al. 2019
Micropropagation + drought stress	Mannitol	Downregulation of several genes encoding key enzymes of the steviol glycoside biosynthetic pathways SvGls decrease	Pandey and Chikara 2015
	Proline	SvGls increase	Gupta et al. 2016
	PEG	SvGls increase	Gupta et al. 2016
Callus and cell suspension culture	plant growth regulators	minor and varied amounts of SvGls	Swanson et al. 1992, Bondarev et al. 2019, 2001 Janarthanam et al. 2010,
Callus and cell suspension culture + salinity stress	NaCl, Na_2_CO_3_	SvGls increase	Gupta et al. 2014
Callus and cell suspension culture + drought stress	Proline, PEG	SvGls increase	Gupta et al. 2015
Transformation with *Rhizobium rhizogenes*, hairy roots production		SvGls not found	Yamazaki and Flores 1991
	Light and dark conditions	Photosynthetically dependent SvGls synthesis and upregulation of *UGT85C2* gene	Pandey et al. 2016
	Light stress and osmotic stress	SvGls synthesis	Libik-Konieczny et al. 2020
Transformation with *Rhizobium rhizogenes*, *Agrobacterium* Mediated Transient Gene Silencing (AMTS)		Identification of alternative pathway of SvGls biosynthesis	Guleria and Yadav 2013a
Transformation with *Rhizobium rhizogenes*, hairy roots production and plantlets regeneration		SvGls increase in regenerated plantlets	Sanchéz-Cordova et al. 2019
Genetic modification of microorganisms	*Escherichia coli*	Reconstruction and expression of kaurene biosynthetic pathway, development of pathways for rebaudioside D synthesis and UDP-glucose recycling, redesigning and reconstruction a steviol-biosynthetic pathway and overproduction of steviol in *Escherichia coli*	Kong et al. 2015, Wang et al. 2015, Chen et al. 2018, Moon et al. 2002
	*Saccharomyces cerevisiae*	De novo production of rare steviol glycosides like Reb M or Reb D	Patents of Washington University, University Massachusetts, Evolva cited in: Spakman 2015
Chemical synthesis/enzymatic synthesis		Rebaudiosides A, D and M chemical synthesis, enzymatic modifications to improve the sweet-tasting of SvGls	Qiao et al. 2018, Gerwig et al. 2016

### Micropropagation

In vitro plant culture is helpful for overcoming many of the limitations of conventional propagation methods in stevia (self-incompatibility and low seed viability and vigour). In order to achieve a genetically uniform plant material with more predictable and homogeneous content of SvGls, micropropagation techniques have been performed (Fig. 5). However, there are discrepancies in the results concerning the total content of SvGls of micropropagated plants with some reports claiming that that SvGls level is not affected by the method of propagation (Kumari and Chandra 2015), while others reporting about 5 times less SvGls in in vitro culture (Bondarev et al. 2001). Considering time as an important factor for the large-scale production of plantlets, micropropagation seems to be irreplaceable. Yücesan et al. (2016) have optimized an efficient micropropagation protocol for the rapid multiplication of *S. rebaudiana* plants through which more than half million of plants can be produced from the single node within 6 months.

#### Elicitors, drought or salinity stress application during micropropagation

Some elicitors like alginate, casein hydrolysate, pectin, yeast extract, methyl jasmonate, salicylic acid or chitosan have been used to increase SvGls content in micropropagated *S. rebaudiana* plants (Bayraktar et al. 2016). These attempts have demonstrated a positive impact of alginate and yeast extract on stevioside level in the micropropagated plantlets. Stevia plants growing in vitro have also been studied for their adaptability to some abiotic stress factors such as salinity or water stress (Pandey and Chikara 2015; Gupta et al. 2016; Fallah et al. 2017; Rameeh et al. 2017; Lucho et al. 2019). Results found in such studies may have potential practical applications for breeding lines that are more tolerant to different types of stressors and exhibit optimal concentration and profile of SvGls as a result of metabolic changes caused by stress factors. However, further research is needed in order to apply optimal concentration of stress factors to metabolic manipulation and SvGls biosynthesis.

### In vitro culture of cells, callus or adventitious roots

**Fig. 6:**
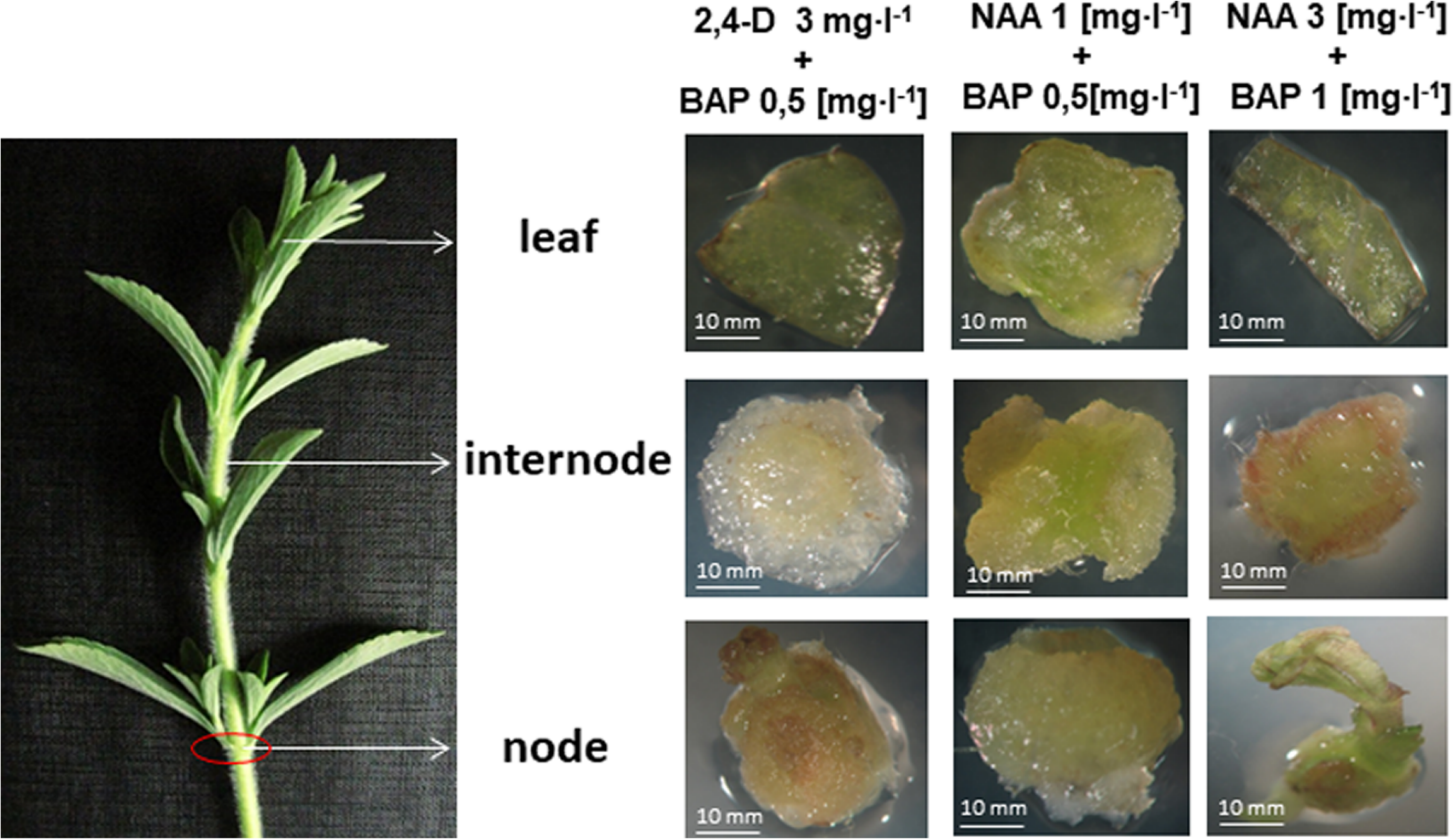
Induction of callus culture from different explants of *S. rebaudiana* cultured on media supplemented with different plant growth regulators (source: unpublished photos by Marta Libik-Konieczny)

Supplementing culture media with plant growth regulators in different types of stevia explants has resulted in a production of callus and cell suspension cultures of *S. rebaudiana* (Swanson et al. 1992; Bondarev et al. 1998; Janarthanam et al. 2010; Gupta et al. 2015; Bondarev et al. 2019) (Fig. 6). These types of cultures have been shown to synthesize only minor amounts of the SvGls, and their content has varied greatly during the growth cycle of the culture. The qualitative composition of the SvGls in the cell cultures appears to be highly scant as compared with that of the donor plants (Bondarev et al. 2001). It has been found that the combination of growth regulators in the medium is an important factor for proliferation of isolated cells of *S. rebaudiana*, while the level of SvGls accumulation depends to a greater extent on genotypic features of cell strain (Bondarev et al. 2019). The limited capability of calli and cell suspensions to synthesize steviol glycosides has been suggested to be closely related to their level of differentiation. A correlation between organelle differentiation and the capacity for steviol glycoside biosynthesis has been underlined with special emphasis on differentiation to mature chloroplasts that appear to be crucial for steviol glycoside biosynthesis to have a maximum output (Ceunen and Geuns 2013a).

Recently, a new approach has been described to synthesize stevioside, rebaudioside A, and dulcoside A by adventitious roots of *Stevia rebaudiana* cultured in media supplemented with gibberellic acid (Ahmad et al. 2020). The novelty of these studies is that adventitious roots are induced from the roots of seedlings grown in vitro and, as it is known so far, SvGls are not produced by the root cells. In earlier studies on establishment of adventitious roots production from *S. rebaudiana* under auxin supplementation, the presence of any known steviol glycosides has not been detected in this type of cultures (Reis et al. 2011).

#### Drought or salinity stress application during in vitro culture

Although some experiments with application of drought or salinity stress factors during in vitro stevia cell cultures have been performed in order to enhance SvGls content, they have never resulted in an increase of these compounds to levels found in the explants (Gupta et al. 2014; Gupta et al. 2015).

### Genetic manipulation

#### Hairy roots production and organogenesis induction from transformed tissue

**Fig. 7:**
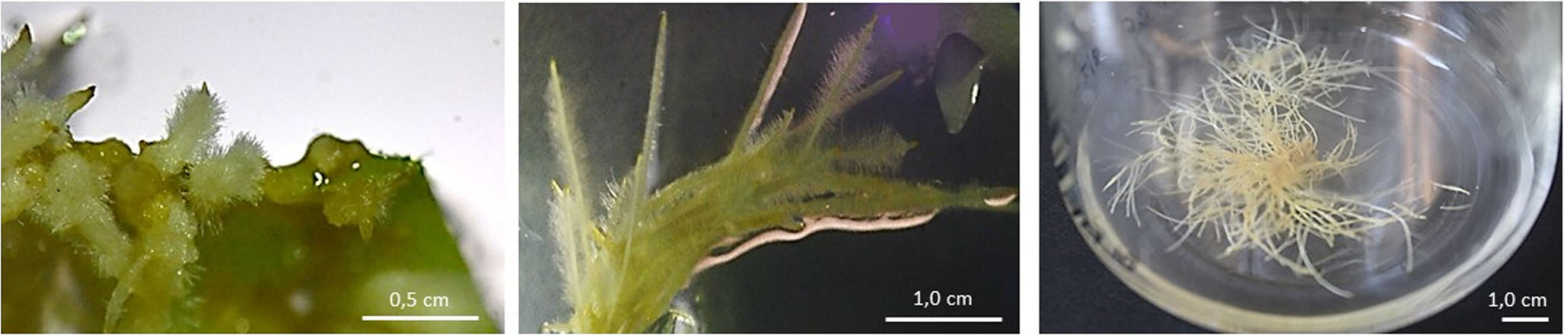
Following steps of hairy roots formation after transformation of *S. rebaudiana* leaf explants with *R. rhizogenes* (source: unpublished photos by Marta Libik-Konieczny)

Due to the unusual ability to biosynthesize compounds naturally produced in the native plant, in vitro techniques for hairy roots (HR) formation from many medicinal plants are utilized for large-scale production of secondary metabolites with high pharmaceutical properties. *Stevia rebaudiana* Bertoni has also been the object of these studies (Fig. 7) (Yamazaki and Flores 1991; Guleria and Yadav 2013a; Michalec-Warzecha et al. 2016; Fu et al. 2015; Pandey et al. 2016; Sanchéz-Cordova et al. 2019; Libik-Konieczny et al. 2020). HR are a result of *Rhizobium rhizogenes* transformation of plant cells. It has been discovered that bacterial genes, *rolA*, *rolB*, *rolC* and *rolD* from Ri plasmid of *R. rhizogenes*, apart from being responsible for the expression of hairy roots phenotype (White et al. 1983), are also potential activators of secondary metabolism in transformed cells. In the study concerning the first attempt to produce HR by stevia plants, SvGs have not been detected in the transformed tissue (Yamazaki and Flores 1991). In the studies by Pandey et al. 2016, the presence of stevioside has not been noted in either HR or in the culture medium; however differences in the ability of stevioside production have been noted between hairy root clones growing in the same conditions. On the other hand, in a recently published report (Libik-Konieczny et al. 2020), the production of different SvGls in HR cultured under all tested conditions has been observed; however their profile depends on the level of oxidative stress in HR tissues and applied stress factor in the culture medium. Because of these discrepancies, the technique for production of transgenic HR cultures as a source of secondary metabolites from *S. rebaudiana* requires better development and more scientific attention. HR culture is an even more promising method not only for mass production of secondary metabolites in in vitro cultures but also as a primary source of material for scientific studies on SvGls biosynthesis via genetic manipulation of genes involved in this pathway. Guleria and Yadav (2013a) employed *Rhizobium*-mediated transformation with RNA interference (RNAi) system to block the synthesis of KA13H and UGT85C2, UGT74G1 and UGT76G and discovered an alternative pathway of SvGls biosynthesis leading to the formation of rubusoside and 10-O-β-glucopyranosyl steviol. Genetic transformation in stevia to alter the expression of desired genes has become more feasible once direct organogenesis protocol from stevia leaf was successfully established by Sreedhar et al. (2008) and Sanchéz-Cordova et al. (2019) and an indirect organogenesis developed by Aman et al. (2013). Recently, Sanchéz-Cordova et al. (2019) have described that transformed *S. rebaudiana* plantlets show higher yield of SvGls production in comparison to the wild type.

#### Genetic modification of microorganisms

Another approach, regarded as a promising alternative, is SvGls production by genetically modified microorganisms. The studies of *Escherichia coli* metabolic engineering have resulted in several papers concerning the possibility of p *ent*-kaurene, *ent*-kaurenoic acid, steviol and steviol glycosides synthesis in these microorganisms (Kong et al. 2015; Wang et al. 2015; Chen et al. 2018; Moon et al. 2002). However, the amount of compounds produced in these systems is relatively low, and the optimization of the steviol glycoside biosynthesis pathways is still required. Recently several patent applications have been submitted by university communities (Washington University and University Of Massachusetts), as well as by biotechnological companies (Evolva), concerning the recombinant production of SvGls in *Saccharomyces cerevisiae*, indicating that these eukaryotic cells are preferred as host cells for rare steviol glycosides like Reb M or Reb D production (Spakman 2015).

### Chemical synthesis

A particular challenge for the production of SvGls is the chemical/enzymatic synthesis or modification to produce pure compounds of special interest. The urgent need to develop this method is due to the fact that some SvGls, in particular stevioside, rubusoside and duloside A, have a slight bitterness and astringency, giving a long-lasting, unpleasant metallic aftertaste that partially limits their use for human consumption and thus limits their use in food and pharmaceutical products (Gerwig et al. 2016). To minimize the bitter aftertaste of SvGls, microencapsulation methods with maltodextrin and inulin as encapsulants, as well as flavor enhancers and flavor modifiers are used. Alternatively, an effective option to improve their organoleptic properties could be de novo chemical synthesis and chemical/enzymatic modifications. Despite the efforts already made, the chemical synthesis of these compounds has only been reported sporadically. Recently, Qiao et al. (2018) have published a protocol for the synthesis of rebaudiosides A, D and M. Several enzyme systems have been tested that introduce additional glucose residues as a result of in vitro carbohydrate-enzyme bioengineering. They have been reviewed in detail by Gerwig et al. (2016) and Spakman (2015). However, due to a lack of knowledge of the structure-sweetness relationships, it is difficult to develop simple protocols for enzymatic synthesis or modification of SvGls. Therefore, many more systematic studies are needed to assess the specificity of glycosylation and its effects on the taste properties of SvGls (Gerwig et al. 2016). It should also be emphasized that the industrial application of chemical/enzymatic methods to synthesize or modify SvGls can be problematic. The use of toxic chemical reagents can lead to acceptance problems in the food industry. To overcome these problems, alternative compounds may be preferred that are more aligned with the goals of “green” chemistry.

## Extraction and purification of steviol glycosides

An important part of obtaining SvGls is their extraction and purification from the plant material in a way that results in a high purity of unchanged compounds.

As a preliminary step, dehydration or drying of plant material is required to prevent growth of microorganisms and changes of biochemical characteristics. Common methods for dehydration include freeze drying, convective drying, vacuum drying, microwave-drying, infrared-drying, sun drying and shade-drying. The drying conditions applied in fresh stevia leaves have a great impact on the extraction of total and particular SvGls. It has been suggested that the least aggressive treatment for all the types of SvGls is shade drying (Periche et al. 2015). However, shade drying can increase the risk of contamination and subsequently results in an adverse impact on plant material quality (Wang et al. 2020). Different conventional and novel technologies have been applied for SvGls extraction including maceration and heat extraction, high temperature and high pressure, electrical voltage, radiation, ultrasound and chromatographic techniques. They have been reviewed in Bursać Kovačević et al. (2018), Castro-Muñoz et al. (2020) and Wang et al. (2020). The extraction yields achieved using these technologies are relatively low varying between 2 and 35%, depending on the solvent and technique used (Castro-Muñoz et al. 2020). These low percentages and large amounts of solvents that have to be removed further downstream in the purification process make it difficult to apply these technologies in large-scale production. Currently, membrane-based technologies are indicated as the most promising tool for SvGls extraction (Castro-Muñoz et al. 2020). These techniques are still being developed, and they include microfiltration, ultrafiltration and nanofiltration. A typical pressure-driven membrane process uses a semipermeable membrane that acts as a selective barrier for the transport of compounds contained in a solution. The advantages of membrane processes include low energy consumption, less extraction time, high separation efficiency, flexibility, high productivity and ease of scaling-up (Castro-Muñoz et al. 2020). Moreover, membrane processes are environmentally friendly since not a lot of chemical solvents are required for efficient extraction. This is of special interest for SvGls due to their intended use in the food and pharmaceutical industries. According to the latest reports, the extraction yields of stevioside and rebaudioside A and their purity, using integrated membrane processes, are in the range of 19–90% and 32–98%, respectively. The value of the extraction yield depends on several parameters, such as operational parameters, intrinsic membrane properties and pretreatment steps (Castro-Muñoz et al. 2020). Last but not least, it is important to consider the possibility of using a “green” solvent to optimize the SvGls extraction. These types of solvents are characterized by low toxicity, easy availability and reusability and high efficiency. Moreover, due to legislative considerations and the changing approach to environmental issues, creation of “green” solvents for the extraction of important trade compounds is becoming an increasingly important area of research (Byrne et al. 2016; Castro-Muñoz et al. 2020). Although with the SvGls it appears that an universal solvent (i.e. water) is sufficient for their extraction, it cannot be ruled out that other types of “green” solvents could provide benefits in the recovery of these compounds.

## Concluding remarks

The sweetness of stevia comes from its leaves which produce steviol glycosides (SvGls) — the natural plant sweeteners. Because of their benefits to human health (e.g. antidiabetic properties, lowering blood pressure, strong radical scavenging activity) and also high taste quality, the development of effective methods of SvGls production is of high research interest. As a result, many different methods, both conventional and biotechnological, have been developed to increase the yield of SvGls biosynthesis.

Conventional breeding methods fail to meet the continuously increasing demand for steviol glycosides due to lack of high quality planting material. Nowadays, plants with desirable traits are propagated by stem cuttings and tissue culture practices, but this also limits the large-scale production of planting material. Biotechnological methods for SvGls biosynthesis include in vitro plants, organs, tissues and cells culture, transformation with bacterial strains and chemical production. All these techniques offer a possibility of experimental manipulation to improve the yield and quality of SvGls. However, the precise manipulation of SvGls metabolism requires a detailed understanding of the role of different genes and enzymes in biosynthesis of different SvGls and their function in plant physiology. Among the different methods for biosynthesis of SvGls, the genetic transformation-based technologies seem to be of particular importance.

**Fig. 8:**
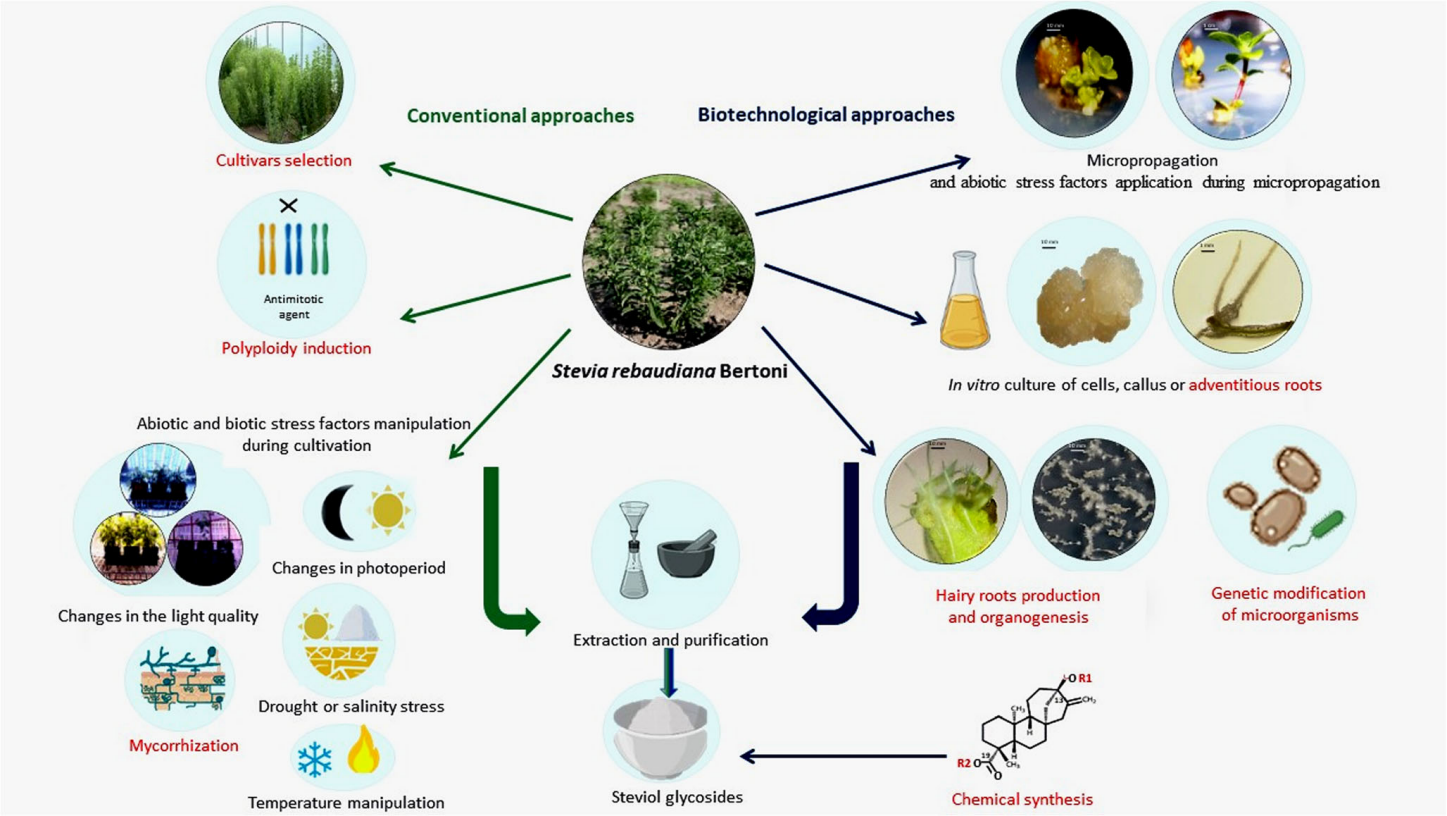
Diagram summarizing various approaches applied for SvGls production. Areas that require further testing have been marked in red font (source: prepared by Marta Libik-Konieczny on the basis of pictures created with BioRender.com and unpublished photos taken by the author)

We hope that the summary presented in this review of previous studies conducted to manipulate SvGls levels (Fig. 8) will help readers to develop future experiments in order to improve the methods of obtaining these valuable secondary metabolites.

## Future challenges

It is imperative to develop economically viable and environmentally sustainable stevia production systems through the integration of site-specific agronomic techniques and efficient mechanization technologies for achievement higher quality product. The choice of the cultivar, propagation and transplanting, sustainable plant management, nutrition, irrigation and harvesting are the aspects that still need to be optimized in order to improve, not only the leaf yield but also its quality.

Recently, autopolyploidization, artificially induced via application of antimitotic agents, appeared as a promising tool for enhanced biosynthesis of plant secondary metabolites in many medicinal plant species (Gantait and Mukherjee 2021). This method should be also considered for investigation of SvGls biosynthesis since still not a sufficient number of experiments in *Stevia rebaudiana* have been performed.

Successful application of protocols for regeneration of transgenic stevia by Sreedhar et al. (2008) and Sanchéz-Cordova et al. (2019) provides an experimental platform for future research on rapid production of plants with modified expression of specific genes targeted to increase the production of SvGls of desired chemical profile.

This is also the case for microbiological biosynthesis, especially that involving unicellular fungi, *Saccharomyces cerevisiae*, which has recently been hailed the most preferable host cell for production of rare SvGls like Reb A or Reb D (Spakman 2015). A direction of research towards production of specific species of SvGls is one of the key opportunities offered by modern biotechnology. However, the complexity of metabolic regulation in plants underlines the difficulties of manipulating the process of transformation via the insertion of one or a few transgenes. Therefore detailed studies are necessary concerning different factors engaged in the induction of particular metabolic pathways. Extensive research involving omics technologies, like transcriptomics, metabolomics and proteomics may provide additional and fundamental information to understand basic chemical processes underlying the control of SvGls biosynthesis and processing. Such advancements will improve the attractiveness of the transformation-based technologies addressing the increase in the yield of SvGls production. It is important to keep in mind, however, that although there is no evidence that foods or food additives derived from transgenic organisms are less safe than non-transgenic ones, there is still a conspicuous degree of public concern about their use. Up until now, SvGls-based sweeteners have been predominantly identified as a plant based product, with pictures of stevia plants or leaves on the packaging of almost all the products. This moral and ethical opposition *per se*, as well as the fear of possible impact of genetic transformation-based technology on food safety, human health and environment must be considered when undertaking such research. In addition, the production of transgenic stevia with high yields of SvGls raises important management problems associated with the cultivation and processing of such plants. One of them, aside from those mentioned above, is development and regulatory approval costs, which in some countries are still relatively high. Standardizing biotechnological procedures, as well as convincing the public about the safety of biotechnology products, will allow for overcoming these barriers and, in consequence, popularizing the use of SvGls.
